# Microfluidic toolbox using padlock probes and rolling circle amplification for direct detection and genotyping of viral RNA

**DOI:** 10.1039/d6ra00912c

**Published:** 2026-04-10

**Authors:** João C. Varela, Priscilla Gomes da Silva, Hower Lee, João R. Mesquita, Aman Russom, Ruben R. G. Soares, Mats Nilsson

**Affiliations:** a Division of Nanobiotechnology, Department of Protein Science, Science for Life Laboratory, KTH Royal Institute of Technology Solna Sweden joao.varela@scilifelab.se; b AIMES (Center for the Advancement of Integrated Medical and Engineering Sciences), Karolinska Institutet, KTH Royal Institute of Technology Stockholm Sweden; c ICBAS-School of Medicine and Biomedical Sciences, University of Porto Porto Portugal; d Epidemiology Research Unit (EPIunit), Institute of Public Health, University of Porto Porto Portugal; e Laboratório para a Investigação Integrativa e Translacional em Saúde Populacional (ITR) Porto Portugal; f Department of Biochemistry and Biophysics, Science for Life Laboratory, Stockholm University Solna Sweden

## Abstract

Rolling circle amplification (RCA) combined with padlock probes presents a promising tool for direct detection and genotyping of viral RNA, offering advantages over conventional methods like RT-PCR. This isothermal process enables highly sensitive and specific amplification of nucleic acids without the need for thermal cycling, making it suitable for point-of-care applications. In this study, we demonstrate a microfluidic and RCA-based method for the direct detection of SARS-CoV-2 RNA and variant profiling, bypassing the reverse transcription step. Our approach allows for the identification of single nucleotide polymorphisms (SNPs) specific to viral variants, enhancing the detection sensitivity through the circle-to-circle amplification (C2CA) technique. This methodology shows potential as a robust, cost-effective platform for viral diagnostics, capable of being fully automated and integrated with miniaturized detection systems for efficient use in both resource-rich and resource-limited settings.

## Abstract 2

Rolling circle amplification (RCA) combined with padlock probes presents a promising tool for direct detection and genotyping of viral RNA, offering advantages over conventional methods like RT-PCR.

## Graphical abstract

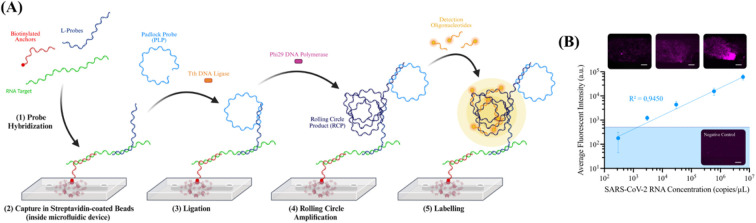

## 1. Introduction

Emerging RNA viruses continue to threaten global public health, as illustrated by the recurring emergence of epidemic and pandemic pathogens over the past century.^1^ The rapid global spread of SARS-CoV-2 further underscored the need for scalable, flexible molecular diagnostics and robust genomic surveillance systems capable of monitoring viral evolution in real time. Genomic surveillance platforms allow early detection of mutations with functional and epidemiological consequences, supporting public health decision-making and outbreak management.^2,3^ However, the global effectiveness of such systems is constrained by resource availability, uneven sequencing capacity, and reliance on centralized infrastructure.^4,5^

PCR remains the most widely used molecular diagnostic method for viral detection due to its high sensitivity and specificity, yet its dependence on thermal cycling, trained personnel, and sophisticated instrumentation limits portability and its applicability to point-of-care (POC) settings.^6,7^ Moreover, variant-specific PCR assays require continuous updating of primer and probe sets as circulating genomes diversify,^8^ whereas sequencing—although definitive—is costly and challenging to deploy universally.^5^ These limitations have accelerated interest in isothermal amplification methods, which operate at constant temperature and are therefore more compatible with decentralized diagnostics.^9,10^

Among isothermal strategies, rolling circle amplification (RCA) is notable for its biochemical simplicity, operational robustness, and programmability. RCA synthesizes long concatemeric DNA products from circular templates and can be coupled to padlock probes (PLPs), whose ligation-based circularization provides single-nucleotide specificity.^11,12^ PLP-RCA has been applied in mutation detection, viral subtyping, and high-plex spatial transcriptomics.^13,14^ RCA's compatibility with microfluidics has further opened opportunities for miniaturized, automated diagnostic platforms offering rapid sample-to-answer workflows. Previous studies have demonstrated femtomolar- to attomolar-level detection of viral nucleic acids—including HIV-1, Zika virus, SARS-CoV-2, and others—through microfluidic bead-based enrichment, integrated photodiodes, and circle-to-circle amplification (C2CA) for signal enhancement.^15–18^

Recent systematic optimization efforts have advanced the integration of PLP-RCA on microfluidic chips by addressing critical design parameters—enzyme concentrations, hybridization strategies, flow conditions, and solid-phase capture chemistries. In a recent study, it was demonstrated that optimized bead-based PLP-RCA workflows coupled with on-chip fluorescence detection can reach limits of detection as low as 0.4 fM, with sample-to-result times of approximately two hours. Their study emphasized that microfluidic RCA performance strongly depends on efficient ligation, amplification residence time, bead-based capture strategies, and minimization of amplicon loss under continuous flow.^19^ These findings highlight the importance of tailored PLP architectures and surface-immobilization schemes for reliable on-chip amplification and support the development of portable, clinically compatible systems—especially in contexts requiring rapid detection of pathogens or antimicrobial-resistance determinants.

Although RCA is naturally suited for DNA targets, recent innovations have significantly improved RNA-templated ligation. Chimeric PLP architectures and RNA-tolerant ligases such as PBCV-1 DNA ligase have increased the fidelity and efficiency of direct RNA detection.^20,21^ Mutation-tolerant PLPs further extend the applicability of the method to hypervariable RNA viruses, enabling resilient performance even in the presence of genomic diversity.^22^ Collectively, these developments position PLP-RCA as a powerful alternative to PCR-based diagnostics—one capable of delivering single-nucleotide resolution without thermal cycling.

Here, we build on these advances to develop an integrated PLP-based RCA platform capable of direct SARS-CoV-2 RNA detection and variant-specific single-nucleotide discrimination, without the need for reverse transcription. By combining hybridization-mediated RCA for direct RNA sensing with C2CA-based genotyping workflows and chimeric padlock probes designed for SARS-CoV-2 mutation profiling, we create a streamlined assay architecture suited for microfluidic automation. This approach demonstrates the potential for low-complexity, rapid, and mutation-resolving detection of viral lineages at the point of care, addressing key limitations of existing PCR-based and sequencing-based surveillance pipelines.

## 2. Methods

### 2.1. Viral RNA samples

Direct viral RNA detection on the microfluidic device using a single round of RCA was performed using synthetic control 2 SARS-CoV-2 RNA (GISAID ID: MN908947.3; Twist Bioscience), supplied at approximately 1 × 10^6^ copies per µL. This material served both as the analytical positive control and as the source for constructing the RT-qPCR standard curve used to quantify viral RNA in experimental samples. For the variant-profiling experiments, TRIzol-inactivated Vero E6 cell culture supernatants containing SARS-CoV-2 were obtained from the Swedish Public Health Agency (Folkhälsomyndigheten), specifically:

• 500 µL TRIzol-inactivated SARS-CoV-2 Beta variant: hCoV-19/Sweden/21-51217/2021.

• 500 µL TRIzol-inactivated SARS-CoV-2 Alpha variant: hCoV-19/Sweden/20-53846/2020.

• 500 µL TRIzol-inactivated SARS-CoV-2 Wuhan strain: SARS-CoV-2/human/SWE/01/2020.

RNA extraction was performed on 500 µL of each supernatant using the Direct-zol RNA Miniprep Plus Kit (Zymo Research) according to the manufacturer's instructions, and RNA was eluted in 50 µL of RNase/DNase-free water. Quantification of viral RNA was conducted using the FoHM-standardized one-step RT-qPCR assay targeting the RdRp gene with the TaqMan™ Fast Virus 1-Step Master Mix (Thermo Fisher Scientific). All reactions were run on a Bio-Rad CFX Real-Time PCR Detection System, and data processing and quantification were performed using the CFX Maestro 1.0 software.

The thermocycling protocol consisted of a reverse transcription step at 50 °C for 15 minutes, followed by an initial denaturation at 95 °C for 2 minutes. Amplification proceeded for 40 cycles, each comprising denaturation at 95 °C for 3 seconds and extension with fluorescence acquisition over 30 seconds at 60–95 °C. To enable absolute quantification, a 10-fold serial dilution standard curve was generated from the Twist Bioscience synthetic SARS-CoV-2 RNA, beginning at 1 × 10^6^ copies per µL and spanning the dynamic range relevant for the samples analyzed. These standards, run in wells designated as standard (Std), were used by CFX Maestro to generate a regression of *C*_q_ values *versus* the log_10_-transformed input RNA quantity. Reaction efficiency and the linearity of the standard curve were evaluated to ensure compliance with accepted analytical thresholds (90–110% efficiency and *R*^2^ > 0.99). Sample viral genome copies were then interpolated from this standard curve based on their measured *C*_q_ values.

The Limit-of-Detection (LoD) of the RT-qPCR assay was calculated automatically by the CFX Maestro software using regression statistics from the standard curve, applying the formula: LoD = (3.3 × SD of regression residuals)/slope.

This calculation identified the lowest RNA concentration reliably distinguishable from background noise while remaining within the validated analytical range of the assay.

### 2.2. Probe design

Different regions of the SARS-CoV-2 RNA genome were targeted for the two different used methodologies.

For the hybridization-based RCA, probes were designed targeting a conserved Orf1ab genomic region encoding for RdRp: three ssDNA capture oligonucleotides containing a biotinylated poly-A tail at the 5′-end (biotinylated anchors) allow the capture of the target in streptavidin-coated agarose beads, while twelve L-shaped ssDNA oligonucleotides (L-probes) targeting this region, have an overhang sequence that enables PLP ligation, as seen in the sequences presented on Table S1.

Variant profiling was performed using a set of six PLPs (two targeting each variant) with a ribose on the 3′-end, targeting 30-bp long sequences in the S-region of the SARS-CoV-2 genome, containing single nucleotide polymorphisms specific for each of the selected variants. These probes were aligned so that the mutation to be detected would be complementary to the 3′-ribose of the PLP, as shown in Table S1. While each variant-specific PLP contained a different detection sequence on the backbone, a primer-hybridization sequence and one restriction motif were added to all the probes, enabling the first and second round of RCA to be performed, respectively. A set of four biotinylated anchors targeting conserved sequences of the S-region were also used to capture the targets in the magnetic streptavidin-coated beads.

All probe sequences were blasted using BLASTn to confirm the specificity and checked for the formation of secondary structures with the mFOLD web tool. All used oligonucleotides were ordered from Integrated DNA Technologies (IA, USA), with their sequences listed in Table S1.

### 2.3. Microfluidic device fabrication and bead packing

Two different flow cell designs, previously described for diagnostic applications, were used in this work: (a) two parallel 600 × 140 µm (width × height) cross-section columns, interconnected by channels with smaller 200 × 20 µm (width × height) cross-sections,^23^ used for the on-chip, hybridization-based RCA; and (b) straight flow cells with a 700 × 100 µm (width × height) cross-section and an interface with a 200 × 20 µm (width × height) cross-section,^24^ used for detecting C2CA amplification products. In both designs, the height difference between the different cross-sections is intended to physically trap the agarose beads at the intersection. A schematic of both designs can be found in Fig. S1.

The microfluidic devices were fabricated in poly(dimethylsiloxane) (PDMS) using standard mold replication and soft lithography techniques in the same way as described in previous works.^23,24^ The pre-polymer was first mixed with curing agent mixture in a ratio of 10 : 1 (w/w) (Sylgard 184, Down Corning), degassed for 30 minutes, poured into the two-level SU-8 mold for the design of choice, and cured at 65 °C for 90 minutes in a convection oven. Access holes were punched after peeling the PDMS from the mold, using a blunt 18-gauge syringe tip, and the microchannels were subsequently sealed against 700 µm thick glass slides (Corning microscope slides, plain) by exposing both surfaces to an oxygen plasma treatment for 30 s (Femto Science CUTE, 100 W, 80 Pa).

Bead packing was performed by flowing a solution of streptavidin-coated agarose beads with an average of 34 µm diameter (Streptavidin Sepharose High Performance, GE Healthcare) mixed in phosphate buffered saline (PBS) (137 mM NaCl, 10 mM phosphate, pH 7.4) at a ratio of 1 : 100 (V/V), at a flow rate of 10 µL min^−1^, by applying negative pressure using a New Era Pump Systems NE-1200 12-channel syringe pump. This step was followed by a wash step with PBS at 10 µL min^−1^ for 2 min, with subsequent sample analysis following afterwards. For design (a), the beads solution was flowed approximately for 2 min, while for design (b) the channel was fully packed with beads. 1% BSA blocking solution in PBS (Thermo Fisher) was also flowed at 5 µL min^−1^ for 10 min right before starting the on-chip RCA experiments.

### 2.4. Hybridization-based rolling circle amplification (HybRCA)

Assay conditions were based on previous published work by our group in which padlock probe ligation and RCA were performed on surfaces and microfluidic channels with different geometries.^25–27^

Hybridization of target SARS-CoV-2 RNA with the respective probes was first performed by mixing 1.5 µL of target with 3 nM of each different L-probes and 10 nM of different biotinylated anchors, in 50 µL of capture solution mix, for a final concentration of 5× SSC, 30% formamide, 0.1 mg mL^−1^ salmon sperm DNA, 1 U µL^−1^ RiboProtect (Blirt, Gdansk), and nuclease-free water. This solution was first incubated at 65 °C for 5 min outside of the chip, without RiboProtect, and subsequently flowed (upon addition of RiboProtect) at 2.5 µL min^−1^ for 15 min, at 45 °C. This was followed by an initial washing step where a washing solution (5× saline-sodium citrate (SSC; Invitrogen); 30% formamide; 0.5% Tween 20) was flowed at 10 µL min^−1^ for 5 minutes, followed by a second washing step with PBS using the same flow conditions and time. PLP hybridization and ligation was performed by flowing the reactional mix containing 10 nM PLPs, 20% formamide, 200 µg µL^−1^ BSA, 1 U µL^−1^ RiboProtect, 0.5 U/µL Tth DNA ligase (Blirt, Gdansk) in the respective ligation buffer (20 mM Tris–HCL pH 8.3, 25 mM KCl, 10 mM MgCl_2_, 0.5 mM NAD, and 0.01% Triton X-100), at a flow rate of 0.5 µL min^−1^ for 30 min, at 45 °C. Upon ligation, amplification was carried out by flowing a second reaction mix containing 5% glycerol, 250 µM dNTPs, 200 µg µL^−1^ BSA, 1 U µL^−1^ RiboProtect, 1 U µL^−1^ phi29 DNA polymerase (Blirt, Gdansk) in 1× polymerase buffer (50 mM Tris–HCL pH 8.3, 10 mM MgCl_2_, and 10 mM (NH_4_)_2_SO_4_), at a flow rate of 0.5 µL min^−1^ for 90 min, at 37 °C. The labeling of resulting RCPs was performed by flowing the final reaction mix consisting of 2× SSC, 20% formamide and 0.1 µM of detection oligonucleotides with a fluorophore (Cy5) at a flow rate of 5 µL min^−1^ for 10 min, at room temperature, followed by a washing step with a solution similar to the last reaction mix, without any detection oligonucleotides, at a flow rate of 10 µL min^−1^ for 5 minutes.

### 2.5. Ligation of PLPs to RNA and circle-to-circle amplification (C2CA)

RNA samples were first hybridized with the respective biotinylated anchors by mixing 2 µL of the target with 100 nM of each biotinylated anchor in a solution comprised of 5× SSC, 0.5% Tween 20, and 1 U µL^−1^ RiboProtect, for a total of 20 µL. This mixture was incubated for 5 min at 50 °C, followed by a 20 min incubation time at room temperature, with 5 µL of streptavidin-conjugated magnetic beads (Dynabeads T1, Thermo Fisher) after these are washed three times with WTW buffer (10 mM Tris–HCl, pH 7.5, 5 mM EDTA, 0.1% Tween 20, 100 mM NaCl). Following this step, the beads were washed once again with WTW buffer, followed by a wash with PBS, and then incubated with a PLP-hybridization mix, consisting of a solution of 1 nM of each PLP, 2× SSC, 5% ethylene carbonate, 15 mM MgCl_2_, 0.1% (V/V) Tween 20 and 1 U µL^−1^ RiboProtect for 30 min at 37 °C. Upon hybridization of the PLPs, the ligation is carried out by removing the supernatant and adding 20 µL of solution containing 5% PEG8000 (Sigma-Aldrich), 0.1 mM ATP, 500 mM Tris–HCl pH 7.5, 10 mM DTT, and 1 U µL^−1^ of homemade T4 RNA ligase 2 enzyme (Protein Science Facility, Karolinska Institutet), followed by incubation at 37 °C for 120 min. Before the first amplification round, 20 µL of solution containing 100 nM of the PLP primer, 2× SSC, 5% ethylene carbonate, 15 mM MgCl_2_, 0.1% (V/V) Tween 20 and 1.25% glycerol was added upon removing the previous supernatant and incubated for 30 min at 37 °C. Amplification was then carried out by once again removing the supernatant and adding 15 µL of the first amplification mix, consisting of 50 mM Tris–HCL pH 8.3, 10 mM MgCl_2_, 10 mM (NH_4_)_2_SO_4_, 5% glycerol, 0.25 mM dNTPs, 0.2 mg mL^−1^ BSA, 0.5 U µL^−1^ Phi29 DNA polymerase (Blirt, Gdansk), and 1 U µL^−1^ RiboProtect, followed by an incubation at 37 °C for 60 min, with a subsequent enzyme inactivation step at 60 °C for 10 min. After the first amplification, 5 µL of restriction solution is added to the solution to enable the second round of amplification. This solution consists of 120 nM of the restriction oligo, 50 mM Tris–HCl pH 8.3, 10 mM MgCl_2_, 10 mM (NH_4_)_2_SO_4_, 0.2 mg mL^−1^ BSA, 800 mU µL^−1^ AluI restriction enzyme (New England BioLabs), and is incubated at 37 °C for 10 min, followed by an enzyme inactivation step at 60 °C for 10 min. Upon digestion, the supernatant is collected and combined with the second amplification mix, for a final concentration of 50 mM Tris–HCL pH 8.3, 10 mM MgCl_2_,10 mM (NH_4_)_2_SO_4_, 0.68 mM ATP, 0.25 mM dNTPs, 0.2 mg mL^−1^ BSA, 0.05 U µL^−1^ T4 DNA ligase (Blirt, Gdansk), 0.5 U µL^−1^ Phi29 DNA polymerase, and incubated at 37 °C for 120 min, with a subsequent enzyme inactivation step at 60 °C for 10 min. Obtained RCPs were then labelled with the respective labelling mix, for a final concentration of 5 nM of each detection oligonucleotide containing the respective fluorophore (Cy3, Cy5 or AF488), as well as 5 nM of a biotinylated oligonucleotide to allow the capture of the amplification product in the streptavidin-coated beads, 2× SSC, 20% formamide, and incubated at 37 °C for 60 min.

### 2.6. Quantification of rolling circle products (RCPs)

For the on-chip hybridization-based direct RNA detection, RCPs were quantified right after the final washing step by imaging the streptavidin-coated agarose beads with an inverted Nikon Ti-Eclipse fluorescence microscope (40× objective) equipped with a SOLA SE light engine and a TRITC, FITC and Cy5 filter cube. The acquired images were then analyzed using the ImageJ software (NIH, USA) by averaging the greyscale intensity in a designated area and comparing it to the fluorescent background on the channel.

RCP quantification for the variant detection experiments was performed as described elsewhere, using both Superfrost® Plus adhesion glass slides (VWR) and the microfluidic chromatography enrichment (µACE), coupled with fluorescence microscopy.^24^ Measurements using the glass slides were performed by placing 10 µL of the labelled RCP solution in the slide, covering it with a cover slip, and imaging it using a Zeiss Axio Imager Z2 epifluorescence microscope (20× objective) equipped with a Cy3, Cy5 and AF488 filter cube. Upon acquiring four independent 300 × 300 µm^2^ images, RCPs were counted using the CellProfiler 4.2.5. software (Broad Insitute, USA), and the sum of the counts per glass slide was determined. For the microfluidic device, one single image of the streptavidin-coated beads was acquired using the aforementioned inverted Nikon Ti-Eclipse fluorescence microscope and its corresponding setting, and RCPs were quantified based on the average grey scale intensity of the bead-packed region, normalized for the fluorescence of the empty channel.

## 3. Results and discussion

### 3.1. On-chip detection of viral RNA using hybridization-based rolling circle amplification (HybRCA)

**Fig. 1:**
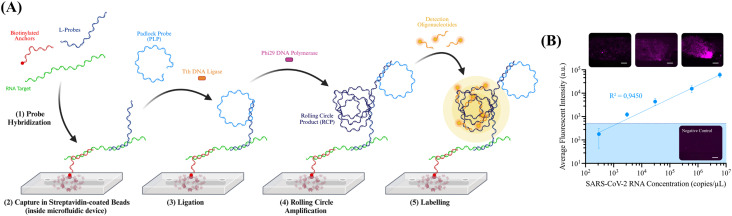
(A) Schematic of the hybridization-based rolling circle amplification (hybRCA), performed entirely inside a microfluidic device by capturing the RNA target in streptavidin-coated agarose beads using biotinylated “capture” oligonucleotides – the biotinylated anchors. (B) Dilution series of SARS-CoV-2 control 2 RNA detected using HybRCA. The shaded region corresponds to the LoD of the method (average of the negative control plus three times the standard deviation; *n* = 3); representative images for 3 detected concentrations (300 copies per µL, 3000 copies per µL and 30 000 copies per µL, respectively); scale bars correspond to 100 µm for each image.

Expanding upon recent developments of RCA-based methods on-chip,^28–33^ we report herein a method for the PCR-free, direct detection of viral RNA on a microfluidic device. For this method, presented in Fig. 1A, the target is (1) hybridized with three biotinylated “anchor” oligonucleotides – allowing the capture of the RNA in streptavidin-coated agarose beads inside the microchannels – and twelve L-shaped probes – containing one sequence targeting the RNA, and an overhang to allow the hybridization of a PLP – all targeting a conserved SARS-CoV-2 region encoding for RdRp. Upon (2) being captured in the respective beads inside the microfluidic device, (3) PLPs targeting the overhang of the L-probes are flowed through the microchannel and, if effectively hybridized, further circularized by the Tth DNA ligase. Upon specific ligation of the PLPs, (4) rolling circle amplification products (RCPs) are generated by the Phi29 DNA polymerase, resulting in one RCP per hybridized L-probe, for an expected total of twelve RCPs per target. Once the amplification is concluded (5) the amplification products are labelled with detection oligonucleotides containing a fluorophore, allowing their detection and quantification through fluorescent microscopy. Due to the multiple steps required to perform the on-chip amplification, it was necessary to confine the beads to prevent their movement during the assay, which could give rise to heterogeneous signals that could hinder the detection performance. To achieve this, we adapted a previously reported diagnostic microfluidic design,^24^ which confines the beads in all spatial directions, thereby preventing movement during the assay and resulting in more homogeneous signal acquisition.

To test the performance of this method in the direct detection of viral RNA for diagnostic purposes and determine its LoD, a dilution series of synthetic SARS-CoV-2 RNA was detected using the aforementioned pool of probes. The LoD was estimated based of the interception of the linear regression of the dilution series with a threshold based on the standard deviation of the blank, negative control experiments containing no RNA, by averaging the registered signal of three separated experiments and adding three times the registered standard deviation. The obtained results, presented in Fig. 1B, show that the on-chip HybRCA is capable of reliably detecting RNA concentrations down to 3000 copies per µL, with an estimated LoD of 1000 copies per µL after 90 minutes of a single round of isothermal amplification. Importantly, this method shows the potential to provide a qualitative “yes/no” answer for a patient with active infection while making use of a sub-cm-sized, robust microfluidic platform. Furthermore, the fast amplification combined with the simple and easily achievable isothermal requirements of the amplification assay, demonstrates significant potential of this assay in approaching diagnostics in both higher and low to middle-income settings. While the limit of detection of the method is not as low as the RT-qPCR gold standard for detecting viral RNA, this detection limit is comparable to that of the standard, one-round RCA methodologies.^24,34^ However, since this methodology relies mostly on hybridization conditions of the probes, it does not yet allow for single nucleotide specific detection of RNA, since the ligation of the padlock probe is not done directly on the target. As such, this technology is particularly directed for point-of-care viral diagnostics that do not the profiling of pathogen variants characterized by their single nucleotide polymorphisms.

### 3.2. Direct viral RNA genotyping resorting to circle-to-circle amplification (C2CA)

RNA detection of pathogens typically requires a reverse transcription step that tends to increase the cost and complexity of the analysis. For PLP-RCA, this is especially problematic, since the lack of highly specific and efficient enzymes that can ligate the PLP in a DNA–RNA interface jeopardizes either the single nucleotide specificity inherently dependent on the proper ligation of the PLP, or the time necessary for the assay to take place, limiting its point-of-care applicability. Ligases typically used for the direct detection of RNA using RCA and PLPs include the Chlorella virus PBCV-1 DNA ligase (SplintR) and the T4 RNA ligase 2 (T4Rnl2). SplintR has been described as capable of ligating RNA-templated ssDNA with very high processivity but relatively poor specificity, typically requiring careful probe design or extra steps to ensure a high ligation accuracy.^35,36^ On the other hand, T4Rnl2 has been shown to provide great ligation specificity when combined with the use of “chimeric” PLPs – a probe with a ribose at the 3′-end instead of a deoxyribose – especially when the probe targets the mutation at its 3′-end. However, the processivity of this enzyme is much lower compared to SplintR, resulting in increased assay times that may not result in enough amplification products to ensure the detection of low target concentrations.^36^

**Fig. 2:**
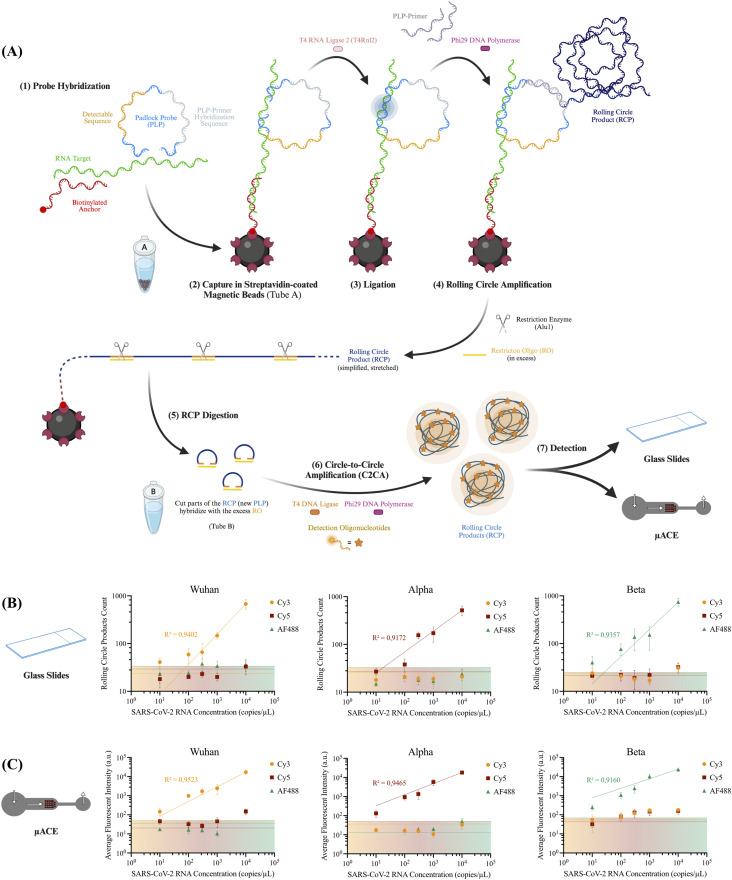
(A) Schematic of the circle-to-circle amplification (C2CA) method used for RNA detection and variant profiling, using T4 RNA ligase 2 (T4Rnl2) as the RNA ligase of choice, combined with “chimeric” padlock probes (PLPs) in the first amplification round. Detection oligonucleotides (DO) with different fluorophores were used to detect the different SARS-CoV-2 variants: Cy3 for Wu (Wuhan strain); Cy5 for Alpha (variant B.1.1.7); and AF488 for beta (variant B.1.351). (B) Counts of amplification products (RCPs) labelled with each of the different used DOs when detecting various concentrations of each SARS-CoV-2 variant. Each value corresponds to the sum of four independent 300 × 300 µm^2^ images of 10 µL of labelled solution in positively charged glass slides. (C) Average fluorescent intensity (au) measured in the microchannels of the µACE for the different used DOs when detecting the various SARS-CoV-2 variants. Each value corresponds to the average grey scale intensity of the bead-packed region, normalized for the fluorescence of the empty channel. The shaded region corresponds to the determined LoD.

By performing two consecutive rounds of amplification, C2CA increases the sensitivity of the assay up to 1000 times per hour on the second round of amplification when compared to single-round RCA,^24,37^ helping to address the T4Rnl2 low processivity in the direct detection of RNA. Aiming to better understand the performance of C2CA when detecting RNA using T4Rnl2 in the first amplification round, dilution series of three individual SARS-CoV-2 variants (Wuhan strain – Wu; B.1.1.7 variant – Alfa; B.1.351 variant – Beta) were detected using a set of six “chimeric” PLPs, with two probes targeting each variant. By targeting each individual variant using this set of probes, it is also possible to better understand if there is any cross-reactivity between any probes and/or targets, as this would result in the detection of non-specific signal in other fluorescent channels. A schematic of the used methodology is found in Fig. 2A. For this method, the calculated LoD was determined by intercepting the linear regression of each dilution series with the highest threshold calculated for the negative control experiments containing no RNA in each fluorescent channel, averaging the registered signal of three separated experiments and adding three times the registered standard deviation. If the linear regression does not intercept this threshold for the tested concentrations, this value is extrapolated based on the equation obtained for the linear regression. This is represented through a shaded-colored area in the results section of Fig. 2(B and C).

The results, summarized in Fig. 2B for the analysis performed in the glass slides and Fig. 2C when using µACE, shown a reliable detection of each individual variant down to a concentration of 100 copies per µL, with a LoD close to 10 copies per µL for all the variants. When comparing the performance of the traditional glass slide RCP counting with the µACE technology, we once again validate the enhanced performance of the microfluidic device demonstrated in our previous work,^16,17^ as the signals registered for the lowest tested concentrations are above the calculated negative control threshold, indicating lower concentrations could potentially be detected, which might not be possible using the glass slide method. By concentrating the amplification products in one small area, a more reliable quantification can also be achieved.^38,39^ The compact geometry of the µACE platform and its ability to concentrate amplification products within a confined detection zone improve quantification reliability compared to conventional glass-slide counting. These features highlight the analytical advantages of microfluidic enrichment for fluorescence-based detection of RCPs. Furthermore, the obtained detection limit matches previously determined values when using the same method for DNA detection, both when quantifying the amplification products through discrete counting using glass slides,^24,40^ and when determining the average fluorescent signal present in the streptavidin-coated beads inside the µACE,^24^ albeit with a devi in the overall signal. It should be noted, however, that although no significant mismatches were observed in these experiments, T4Rnl2 has been described has being more specific and efficient when ligating certain nucleotides compared to others.^36^ As such, this should be considered when designing future probes for targeting other mutations, wherein shifting the ligation site 1–2 bp away from the mutation can often be sufficient to circumvent this limitation.

### 3.3. Detection of multiple viral strains – variant profiling using C2CA

To further test the capability of C2CA in detecting multiple single nucleotide polymorphisms specific to the three used viral variants, mixed solutions containing various combinations and concentrations of each variant were detected using this methodology. Quantification was done once again using both positively charged glass slides for discrete RCP counting, and µACE for determining the average fluorescent signal of the amplification products.

**Fig. 3:**
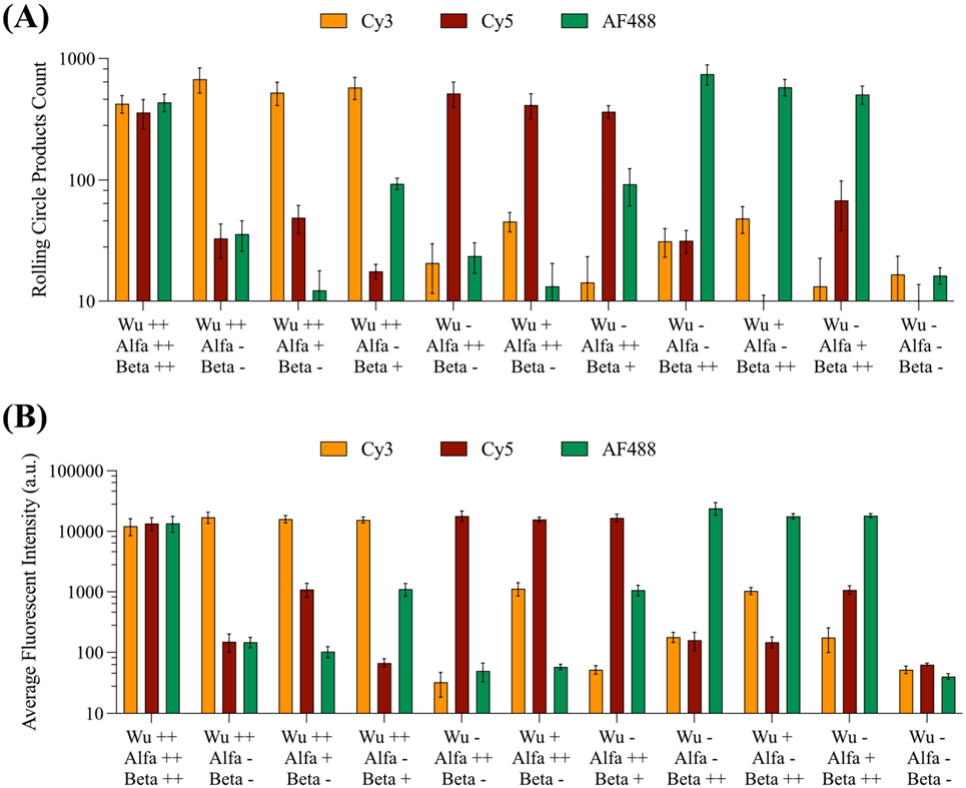
Registered signal obtained for each different DO when detecting various samples containing various concentrations and SARS-CoV-2 variants using (A) positively charged glass slides or (B) µACE as a detection method. The solutions contain either (−) none of the variant, (+) 10^2^ copies per µL of the variant, or (++) 10^4^ copies per µL of the variant (*n* = 3).

The results presented in Fig. 3 highlight that T4Rnl2 enables the specific direct detection of each different RNA, while the quantified amplification products (counts in the glass slides, Fig. 3A; average fluorescent signal, Fig. 3B) allow the estimation of the amount of RNA present in each solution. Importantly, this demonstrates the potential of C2CA in not only detecting RNA directly through the use of “chimeric” PLPs and T4Rnl2 in the first ligation step, but also in currently identifying different single nucleotide polymorphisms between different viral strains.

## 4. Conclusion

In this study, we present two complementary PLP-RCA–based strategies for the direct detection and genotyping of viral RNA integrated within microfluidic platforms. The hybridization-based RCA (HybRCA) enables PCR-free detection of SARS-CoV-2 RNA down to 10^2^ to 10^3^ copies per µL using a single amplification round, while the C2CA workflow permits single-nucleotide discrimination and variant profiling down to approximately 10 copies per µL. Together, these results demonstrate that direct RNA-templated ligation combined with isothermal amplification can achieve analytically relevant sensitivity without reverse transcription or thermal cycling.

Despite these advances, several technical limitations remain. First, amplification times (90–120 minutes per round) remain longer than RT-qPCR and may constrain rapid diagnostic deployment. Second, RNA-templated ligation efficiency is enzyme-dependent, and T4Rnl2 exhibits nucleotide bias that may affect probe design flexibility for certain mutations. Third, although the microfluidic enrichment platform improves signal concentration and quantification, full sample-to-answer integration—including RNA extraction, fluidic automation, and detection—has not yet been demonstrated in a closed, field-ready system. Additionally, fluorescence-based readout currently relies on laboratory-grade microscopy, which limits immediate portability.

Future development should therefore focus on (i) reducing amplification time through optimization of enzyme kinetics and signal chemistry, (ii) integrating upstream sample preparation steps into a fully automated microfluidic cartridge, and (iii) implementing compact optical detection modules compatible with portable or low-cost imaging systems. The demonstrated compatibility of PLP-RCA and C2CA with microfluidic confinement provides a clear engineering pathway toward such integration.

Importantly, this work establishes the first demonstration of direct RNA detection combined with PLPs and C2CA for signal amplification within a microfluidic format. By achieving mutation-resolved viral RNA detection without reverse transcription, this platform offers a promising alternative to PCR- and sequencing-based approaches for decentralized molecular diagnostics and pathogen surveillance.

## Conflicts of interest

The authors declare no conflicts of interest.

## Supplementary Material

RA-016-D6RA00912C-s001

RA-016-D6RA00912C-s002

RA-016-D6RA00912C-s003

RA-016-D6RA00912C-s004

## Data Availability

All essential data supporting the conclusions of this study are included in the main text or provided in the supplementary information (SI). Supplementary information is available. See DOI: https://doi.org/10.1039/d6ra00912c.
