# Supplementary material for: Microfluidic toolbox using padlock probes and rolling circle amplification for direct detection and genotyping of viral RNA
Source: RSC Adv. 2026 Apr 10;16(21):19119–27. doi: 10.1039/d6ra00912c (PMC13068080; doi:10.1039/d6ra00912c)
Supplement: RA-016-D6RA00912C-s002 [file RA-016-D6RA00912C-s002.pdf]

Supplementary Information (SI) for RSC Advances.
This journal is © The Royal Society of Chemistry 2026

Supporting Information

Microfluidic Toolbox using Padlock Probes and Rolling Circle Amplification for Direct Detection and Genotyping of viral RNA

João C. Varela[1, 2†*], Priscilla Gomes da Silva[3, 4, 5†], Hower Lee[6], João R. Mesquita[3, 4, 5], Aman Russom[1, 2], Ruben R. G. Soares[6], Mats Nilsson[6]

[1]Division of Nanobiotechnology, Department of Protein Science, Science for Life Laboratory, KTH Royal Institute of Technology, Solna, Sweden

[2]AIMES Center for the Advancement of Integrated Medical and Engineering Sciences at Karolinska Institutet and KTH Royal Institute of Technology, Stockholm, Sweden

[3]ICBAS-School of Medicine and Biomedical Sciences, University of Porto, Porto, Portugal

[4]Epidemiology Research Unit (EPIunit), Institute of Public Health, University of Porto, Porto, Portugal

[5]Laboratório para a Investigação Integrativa e Translacional em Saúde Populacional (ITR), Porto, Portugal

[6]Department of Biochemistry and Biophysics, Science for Life Laboratory Stockholm University, Solna, Sweden

†These authors contributed equally to this work; *E-mail: joao.varela@scilifelab.se

Table S1: List of biotinylated capture oligonucleotides (Biot-Anchor), padlock probes (PLP), L-shaped oligonucleotides (L-Probe), detection oligonucleotides (DO), PLP-primer and restriction oligonucleotide (RO) sequences used for each assay. Color codes highlight complementary regions, with green highlighting sequences complementary to the target, blue for complementary probes, and red for detection probes. Riboses are identified in the "chimeric" PLPs with a purple color.

**Sequence ID** (assay)	**Sequence (5' - 3')**
**Biot-Anchor 1** (HybRCA)	**Biotin - AAAAAAAAAA TAGTGAACCGCCACACATGACCATTT CACTCAATACTTGAGCACACTCAT**
**Biot-Anchor 2** (HybRCA)	**Biotin - AAAAAAAAAAAA TGTTTACGCAAATATGCGTAAAACT CATTCACAAAGTCTGTGTCAACA**
**Biot-Anchor 3** (HybRCA)	**Biotin - AAAAAAAAAA GCTTCAGACATAAAAACATTGTTTTGAT AATAAAGAACTGACTTAAAGTT**
**L-Probe 1** (HybRCA)	**ACATGTTGTGCCAACCACCATAGAATTTGCT TTTTAGTGT TACGATGTTGACCCCTGTA TTAGGGATGCGGGTAGTA**
**L-Probe 2** (HybRCA)	**CATTTGAGTTATAGTAGGGATGACATTACGT TTTTAGTGT TACGATGTTGACCCCTGTA TTAGGGATGCGGGTAGTA**
**L-Probe 3** (HybRCA)	**CAGATAGAGACACCAGCTACGGTGCGAGCTC TTTTAGTGT TACGATGTTGACCCCTGTA TTAGGGATGCGGGTAGTA**
**L-Probe 4** (HybRCA)	**GCCATAATTCTAAGCATGTTAGGCATGGCTC TTTTAGTGT TACGATGTTGACCCCTGTA TTAGGGATGCGGGTAGTA**
**L-Probe 5** (HybRCA)	**GCTACAACACGTTGTATGTTTGCGAGCAAGA TTTTAGTGT TACGATGTTGACCCCTGTA TTAGGGATGCGGGTAGTA**
**L-Probe 6** (HybRCA)	**CAATTACTACAGTAGCTCCTCTAGTGGCGGC TTTTAGTGT TACGATGTTGACCCCTGTA TTAGGGATGCGGGTAGTA**
**L-Probe 7** (HybRCA)	**CCCAACCCATAAGGTGAGGGTTTTCTACATC TTTTAGTGT TACGATGTTGACCCCTGTA TTAGGGATGCGGGTAGTA**
**L-Probe 8** (HybRCA)	**GTGGCATCTCCTGATGAGGTTCCACCTGGTT TTTTAGTGT TACGATGTTGACCCCTGTA TTAGGGATGCGGGTAGTA**
**L-Probe 9** (HybRCA)	**GACACTCATAAAGTCTGTGTTGTAAATTGCG TTTTAGTGT TACGATGTTGACCCCTGTA TTAGGGATGCGGGTAGTA**
**L-Probe 10** (HybRCA)	**GAAACACACAACAGCATCGTCAGAGAGTATC TTTTAGTGT TACGATGTTGACCCCTGTA TTAGGGATGCGGGTAGTA**
**L-Probe 11** (HybRCA)	**GAGGTCCTTTAGTAAGGTCAGTCTCAGTCCA TTTTAGTGT TACGATGTTGACCCCTGTA TTAGGGATGCGGGTAGTA**
**L-Probe 12** (HybRCA)	**GCCGGCCCCTAGGATTCTTGATGGATCTGGGTTTTAGTGT TACGATGTTGACCCCTGTA TTAGGGATGCGGGTAGTA**
**PLP** (HybRCA)	**TACAGGGGTCAACATCGTA TGCGTCTATTTAGTGGAGCCTT AGTAGCCGTGACTATCGACT ACTACCCGCATCCCTAA**

**Sequence ID** (assay)	**Sequence (5' - 3')**
**Biot-Anchor 1** (C2CA)	**Biotin - TTTTT AACATTAGTAGCGTTATTAACAA TAAGTAGGGACTGGGTC**
**Biot-Anchor 2** (C2CA)	**Biotin - TTTTT CCAGTTTGCCCTGGAGCGATTTG TCTGACTTCATCACCTC**
**Biot-Anchor 3** (C2CA)	**Biotin - TTTTT GAAACTTTTTGTTAGACTCAGTA AGAACACCTGTGCCTGT**
**Biot-Anchor 4** (C2CA)	**Biotin - TTTTT AGCAGCAAGATTAGCAGAAGCTC TGATTTCTGCAGCTCTA**
**PLP 1-Wu** (C2CA)	**AGTGGGTTGGAAACC GTGTATGCAGCTCCTCAGTAATAGTGTCTTAC TGCGTCTATTTAGTGGAGCC GTAACCAACACCATrU**
**PLP 2-Wu** (C2CA)	**CCTGATAAAGAACAG GTGTATGCAGCTCCTCAGTAATAGTGTCTTAC TGCGTCTATTTAGTGGAGCC CTGTGCAGTTAACArU**
**PLP 1-Alfa** (C2CA)	**CAATGTCTCTGCCAA GTGTATGCAGCTCCTCAGTAATAGTGTCTTAC AGTAGCCGTGACTATCGAC CATCAGTAGTGTCArU**
**PLP 2-Alfa** (C2CA)	**AAGGATATCATTTAA GTGTATGCAGCTCCTCAGTAATAGTGTCTTAC AGTAGCCGTGACTATCGAC TTTGTCAAGACGTGrC**
**PLP 1-Beta** (C2CA)	**CACGCACTAAATTAA GTGTATGCAGCTCCTCAGTAATAGTGTCTTAC AGTCGATAGTCACGGCTACT AACCCTGAGGGAGArC**
**PLP 2-Beta** (C2CA)	**AACACCATTACAAGG GTGTATGCAGCTCCTCAGTAATAGTGTCTTAC AGTCGATAGTCACGGCTACT ACAATTAAAACCTTrU**
**PLP-Primer** (C2CA)	**TACTGAGGAGCTGCATACAC**
**RO** (C2CA)	**GTGTATGCAGCTCCTCAGTA**
**DO Biot-Common** (C2CA)	**GTAAGACACTATTACTGAGGATTTT - Biotin**
**DO Cy3-Wu** (C2CA)	**GGCTCCACTAAATAGACGCATTTT - Cy3**
**DO Cy5-Alfa** (C2CA)	**AGTCGATAGTCACGGCTACTTTT - Cy5**
**DO AF488-Beta** (C2CA)	**AGTAGCCGTGACTATCGACTTTT - AF488**

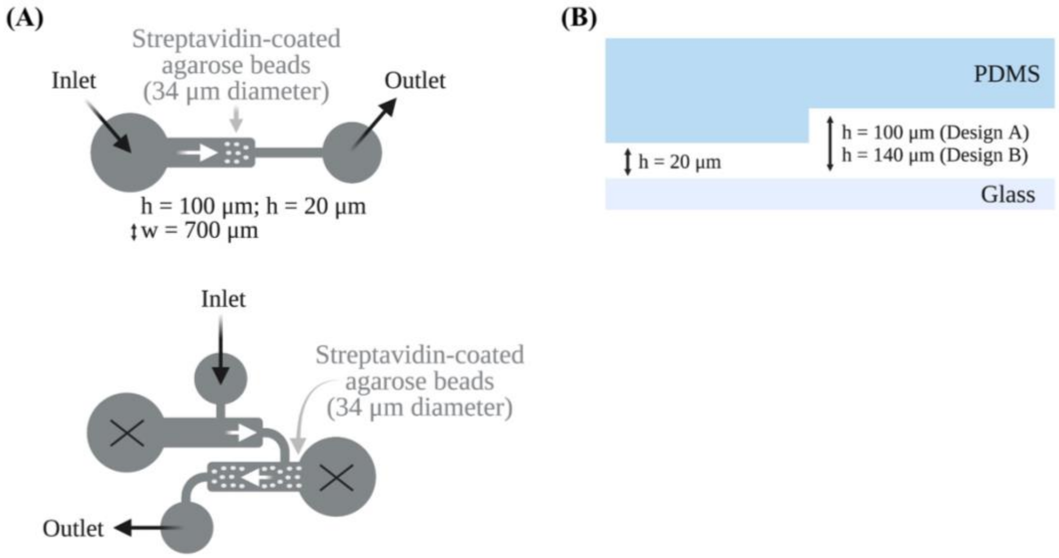

Figure S1: (A) Top and **(B)** side view of the microfluidic device designs. White arrows indicate the direction of the flow.
